# Fat Loss in Continuous Enteral Feeding of the Preterm Infant: How Much, What and When Is It Lost?

**DOI:** 10.3390/nu10070809

**Published:** 2018-06-23

**Authors:** Carlos Zozaya, Alba García-Serrano, Javier Fontecha, Lidia Redondo-Bravo, Victoria Sánchez-González, María Teresa Montes, Miguel Saenz de Pipaón

**Affiliations:** 1 Neonatology Department, La Paz University Hospital, Autonomous University of Madrid, 28046 Madrid, Spain; vickysg85@hotmail.com (V.S.-G.); maitemontesb@gmail.com (M.T.M.); miguel.saenz@salud.madrid.org (M.S.d.P.); 2 Bioactivity and Food Analysis Department, Institute of Food Science Research (CIAL, CSIC-UAM), Autonomous University of Madrid, 28049 Madrid, Spain; albamaria.garcia.serrano@csic.es (A.G.-S.) j.fontecha@csic.es (J.F.); 3 Preventive Medicine and Public Health Department, La Paz University Hospital, Autonomous University of Madrid, 28046 Madrid, Spain; lidiaredondobravo@gmail.com; 4 Carlos III Health Institute, Maternal and Child Health and Development Research Network, 48903 Barakaldo, Bizkaia, Spain

**Keywords:** preterm infant, enteral nutrition, lipids, omega-3 fatty acids, omega-6 fatty acids, Docosahexaenoic acid, Arachidonic acid, long-chain polyunsaturated fatty acids

## Abstract

Human milk fat is a concentrated source of energy and provides essential and long chain polyunsaturated fatty acids. According to previous experiments, human milk fat is partially lost during continuous enteral nutrition. However, these experiments were done over relatively short infusion times, and a complete profile of the lost fatty acids was never measured. Whether this loss happens considering longer infusion times or if some fatty acids are lost more than others remain unknown. Pooled breast milk was infused through a feeding tube by a peristaltic pump over a period of 30 min and 4, 12 and 24 h at 2 mL/h. Adsorbed fat was extracted from the tubes, and the fatty acid composition was analyzed by gas chromatography-mass spectrometry. Total fat loss (average fatty acid loss) after 24 h was 0.6 ± 0.1%. Total fat loss after 24 h infusion was 0.6 ± 0.1% of the total fat infused, although the highest losses occur in the first 30 min of infusion (13.0 ± 1.6%). Short-medium chain (0.7%, *p* = 0.15), long chain (0.6%, *p* = 0.56), saturated (0.7%, *p* = 0.4), monounsaturated (0.5%, *p* = 0.15), polyunsaturated fatty (0.7%, *p* = 0.15), linoleic (0.7%, *p* = 0.25), and docosahexaenoic acids (0.6%, *p* = 0.56) were not selectively adsorbed to the tube. However, very long chain fatty (0.9%, *p* = 0.04), alpha-linolenic (1.6%, *p* = 0.02) and arachidonic acids (1%, *p* = 0.02) were selectively adsorbed and, therefore, lost in a greater proportion than other fatty acids. In all cases, the magnitude of the loss was clinically low.

## 1. Introduction

Fat is an important nutrient for preterm infants [1]. Lipids provide infants most of their energy needs. Lipids also offer specific supplies critical for growth and development like long and very long chain polyunsaturated fatty acids (LCPUFA) including essential fatty acids (Alpha-linolenic and Linoleic acids) and their main derivatives: Docosahexaenoic acid (DHA) and Arachidonic acid (ARA). DHA and ARA seem to be semi-essential for the preterm infant [2]. Both are major components of the brain, and retinal cell membranes and might be related to neurodevelopment and visual function. In case of an early deficit of these fatty acids, there is an increased risk of prevalent preterm morbidities, like sepsis and bronchopulmonary dysplasia [3]. Unfortunately, this early deficit might be common, as current nutritional practices (early parenteral nutrition, with lipid emulsions not designed specifically for the preterm infant) do not deliver the same amount of LCPUFA than a fetus of the same gestational ages would receive in-utero [4].

Human milk (HM) is the recommended diet for all infants, including very low birth weight (VLBW) infants. For the latter, HM is usually delivered via an enteral feeding tube until the preterm infant can be fed orally. Continuous enteral feeding is used in the neonatal intensive care unit as an alternative to bolus/gavage feeding in some clinical scenarios (e.g., enteral intolerance or persistent hypoglycemia) [5]. In 1978, Brooke and Barley reported for the first time that human milk fat delivery was reduced when milk was continuously infused [6]. During the 80’s and 90’s different authors had similar results, reporting total fat losses up to 90% after 8 h of infusion of freshly collected human milk [7]. To limit fat loss, several strategies have been tested (higher infusion flow rates, syringe angulation, milk refrigeration, use of eccentric nozzle syringes, use of peristaltic syringe pumps, previous and frequent milk homogenization, etc.). These interventions proved to be useful to a certain degree [7,8,9,10,11,12,13]. However, concern about fat loss during continuous enteral nutrition is a recurrent issue that still appears to be a problem. Recent research studies have reported total fat losses between 4 and 25% [13,14,15,16]. Whether fat loss is important enough to be clinically relevant in real conditions remains to be clarified. It is important to note that the infusion time of all these studies has been shorter than 8 h, whereas feeding tubes are usually used for longer times in real conditions. Previous reports suggested that lipid losses were not constant over time, but timing of the greater losses (at the beginning of the infusion or later on) is still a controversial issue [6,15,17,18]. Moreover, there are other aspects related to fat loss during continuous enteral nutrition that have not yet been investigated. To date, most reports have focused on total fat losses or have only described what happens to lipid fractions (i.e., triglycerides), but we have no data on possible different losses of individual fatty acids depending on fatty acid characteristics (i.e., chain length or degree of unsaturation). Not all fatty acids have the same biological functions, and some of them are essential in humans or semi-essential for the preterm infant.

We conducted an in vitro experiment, which mirrors in vivo current clinical practice, over a 24-h period. Our objectives were: (1) to determine whether fat losses are constant over the infusion time and, if not, when they are more pronounced over the 24-h period; and (2) to test whether there is a selective loss of individual or groups of fatty acids depending on chain length and degree of unsaturation.

## 2. Materials and Methods

Pooled donor, non-pasteurized HM (1100 mL) was used in this experiment. The HM used in this experiment was progressively collected and was kept frozen for a mean period of 2.5 months (range 1.9–4.7) at −20 °C. It was defrosted before the experiment keeping the sample in refrigeration conditions (5 °C) over 24 h.

The experiment reproduced our standard clinical practices. HM was infused through a 4-French diameter and 40-cm polyvinyl chloride (PVC) di-(2-ethylhexyl) phthalate (DEHP)-free feeding tube (Nutrisafe 2, Vygon, Écouen, France) attached to a PVC system 150 cm in length and 1.5 × 2.5 mm in diameter (Nutrisafe 2, Vygon) by a peristaltic pump (Alaris Enteral, CareFusion, San Diego, CA, USA). The feeding tube and part of the attached system were inside an incubator (Incubator 8000 SC Dräger, Lübeck, Germany). The incubator was set at 33 °C and 60% humidity. The syringe, pump and the rest of the connecting systems were outside the incubator. Average room temperature was 23.8 °C (range 22.7–24.7 °C), and the humidity was 36.9% (range 35–39%). The entire experiment was done within the same 24 h. HM was loaded into 20-mL syringes for enteral nutrition. Then the syringe was hand shaken to homogenize the milk and placed in the pump, with the syringe maintained in a vertical position (tip upwards). Infusions then were programmed at 2 mL/h. When infusion finished, feeding tubes and systems were washed with a distilled water bolus (5 mL) to remove the remaining milk. Then, the feeding tubes and connecting systems were collected and immediately stored at −20 °C. This whole procedure was repeated infusing milk over 30 min, 4, 12 and 24 h, in quadruplicate for each infusion time. To reproduce a real 12 and 24-h infusion, both the HM and the syringes were changed every 4 h. The pooled HM was kept refrigerated (5 °C) during the experiment day. Aliquots (20 mL) were extracted and then left at room temperature for 30 min before filling the syringes, to warm the HM, according to our standard practice. Pre-infusion HM samples were collected at time zero, and at 12 and 24 h from the beginning of the experiment. Following collection, the milk samples were stored at −20 °C.

### 2.1. Total Fat Extraction and GC-MS Analysis

The milk fat adsorbed inside the tubes was extracted using high-performance liquid chromatography-grade hexane as a solvent. More commonly used extraction solutions, such as chloroform 2/methanol 1 (Folch) and hexane/isopropanol (Hara and Radin), were initially used, but they extracted silicones from the tube’s inner surfaces, contaminating the sample and raising concerns about chromatogram reliability. We subsequently verified that hexane allows extraction of the total fat, given no compounds of dairy fatty acids were detected at the retention times when the tubes were ultimately washed according to the Folch [19] or the Hara and Radin method [20]. Blank control tubes (*n* = 3) were washed with the same solvent but without having passed any milk.

The lipid extracts obtained were concentrated by removing the organic solvent under a gentle stream of nitrogen. Then, the lipid extracts were weighed and analyzed as fatty acid methyl esters (FAMEs) obtained by direct derivatization of samples, as described by Castro-Gómez et al. [21]. Briefly, lipid extracts were transferred to borosilicate glass tubes with an acid/heat resistant cap containing 100 μL of tritridecanoin in hexane as internal standard (1.3 mg/mL). Then, 1 mL of 3 M H_2_SO_4_ in methanol was added to each tube and heated for 30 min at 98 °C. After incubation, the samples were cooled in ice for 5 min, and 1 mL of hexane was added. The samples were vortexed for 30 s, and the reaction was then stopped with 7.5 mL of 6% solution of sodium hydrogen carbonate and centrifuged at 1000× *g*, at 4 °C, for 5 min. The upper organic layer containing the FAME was collected and transferred to amber vials for GC–MS injection and 1 μL (at 1:10 split ratio) was injected into a 6890 Agilent gas chromatograph (Palo Alto, CA, USA) fitted with a mass spectrometry (MS) (Agilent 5973 N) detector in a 100-m CPSil-88 capillary column (100 m × 0.25 mm inner diameter × 0.2 μm film thickness (Chrompack, Middelburg, The Netherlands). The GC-MS temperature program and conditions were those previously reported by Rodriguez-Alcala and Fontecha [22]. Briefly, the column was maintained at 100 °C for 1 min after injection and temperature-programmed at 7 °C/min to 170 °C, maintained there for 55 min, and then raised 10 °C/min to 230 °C and maintained there for 33 min. The injector temperature was set at 250 °C. Helium was used as carrier gas with a column inlet pressure of 30 psi. MS detector conditions were a transfer line temperature of 250 °C, a source temperature of 230 °C, a quad temperature of 150 °C and electron impact ionization at 70 eV. For peak identification, mass spectra obtained in our analysis were compared with those in the National Institute of Standards and Technology Library (Gaithersburg, MD, USA). For the qualitative and quantitative analyses, response factors were calculated using anhydrous milk fat (reference material BCR-164) and Supelco 37 FAME mix (Sigma, St. Louis, MO, USA). Tritridecanoin as internal standard (200 μL; 1.3 mg/mL) was also used. Assays were performed in triplicate.

Losses of total fat and fatty acids at each time point were expressed as percentages of the total infused amount recovered from the tube. Percentages were calculated according to the formula:

% Loss = R × 100/(C × V),
(1)

where “R” stands for the raw amount of fatty acid / total fat (mg) recovered from the tube, “C” means milk’s fatty acid concentration (mg/mL) and “V” is the volume infused through the tube over the set time (1 mL over 30 min, 8, 24 and 48 mL over 4, 12 and 24 h, respectively).

A fatty acid was classified as very-long-chain fatty acid (VLCFA) if it had >18 carbon atoms, long-chain fatty acid (LCFA) when it had 16 or 18 carbon atoms and short-medium chain fatty acid (SMCFA) if it had between 6 and 14 carbon atoms.

### 2.2. Statistical Analysis

The statistical analysis was performed using SPSS 20 statistical software (IBM Corporation, Armonk, NY, USA). Descriptive data are presented as mean (± standard deviation) and frequency (%) as appropriate. The Mann–Whitney U test was used to calculate median differences between total fat (which is equivalent to mean fatty acid loss) and individual/families of fatty acid rate losses at 24 h. The Kruskal–Wallis rank test was performed to compare median losses among more than two groups according to the fatty acid composition in various time periods (30 min, 4, 12 and 24 h). A selected one-to-one post hoc analysis was performed correcting significance according to the Dunn-Bonferroni method. Losses of total fat (average fatty acid loss) and individual selected fatty acids over time were analyzed with a curvilinear regression model. For these models, we selected the most abundant fatty acids in the HM or those which seem to be more clinically relevant (essential fatty acids and LCPUFA).

### 2.3. Ethical Issues

The La Paz University Hospital research ethics committee approved the study, and our donors provided informed consent to use their milk for research purposes.

## 3. Results

The fatty acid composition of the pooled HM used in this study is described in Table 1. Samples were analyzed at time zero, 12 and 24 h on the experiment day to rule out oxidative changes affecting the relative fatty acid composition of the HM before it was infused. There were no statistically significant differences regarding saturated fatty acids (SFA), monounsaturated fatty acids (MUFA) and polyunsaturated fatty acids (PUFA) nor regarding chain length (short-medium, long and very long fatty acids) relative composition. Furthermore, individual LCPUFA: alpha-linolenic acid (ALA), linoleic acid (LNA), DHA and ARA concentrations (mg/100 mg of fat) remained stable throughout the study period of 24 h (differences were not statistically significant).

**Figure 1:**
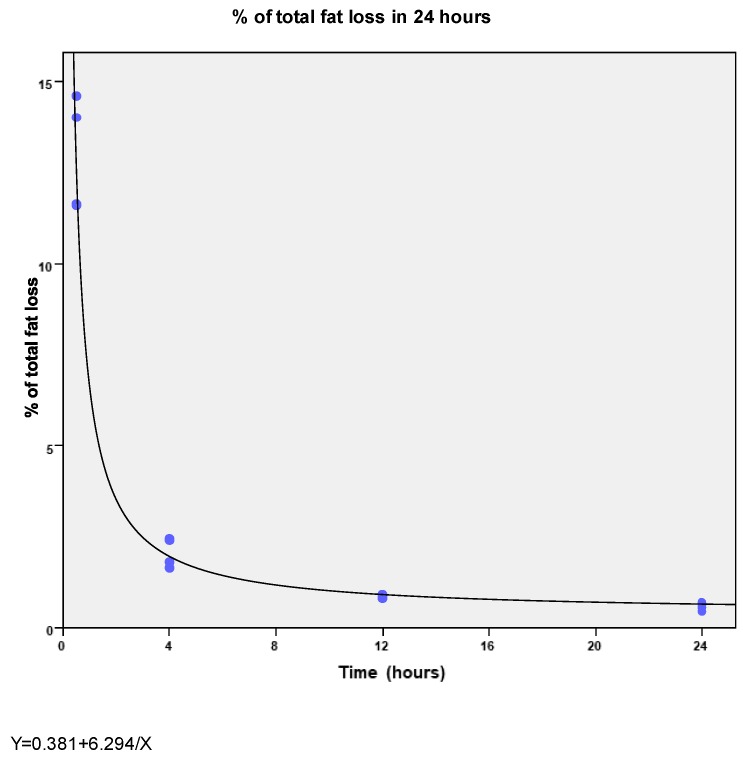
Amount of the total fat loss as % of the fat adsorbed in the tube in relation with the total fat infused during 24 h.

Over 24 h, the mean total fat loss was 0.6 ± 0.1% of the total fat infused. However, fat loss was not constant. The highest fat losses occur in the first 30 min of infusion (13.0 ± 1.6%) and then fat loss progressively decreased at 4 and 12 h to 2% ± 0.4% and 0.87% ± 0.04%, respectively (R^2^ = 0.98; *p* <0.0001) (Figure 1).

Fatty acids were lost in different percentages, at different time points, according to the degree of unsaturation of the fatty acid chain (Table 2). Saturated fatty acids were lost in a higher proportion than monounsaturated fatty acids at 30 min (30.6% vs. 5.8%, *p* = 0.01) and 12 h of infusion (1.1% vs. 0.6%, *p* = 0.02), whereas we found no differences when comparing saturated or monounsaturated with polyunsaturated fatty acids. At 24 h, there were no significant differences among the three groups.

Regarding the length of the chain, fatty acids were lost differently from 4 h of infusion onwards (Table 2). VLCFA loss was higher than that of LCFA at 4 h (3.7% vs. 2%, *p* = 0.02), at 12 h (1.4% vs. 0.8%, *p* = 0.005) and, at 24 h (0.9% vs. 0.6%, *p* = 0.03). We did not find differences between VLCFA and SMCFA or between SMCFA and LCFA at any time.

We also studied the loss of some individual fatty acids. The most abundant fatty acids in the HM (16:0 and 18:1n-9) or those which are especially relevant for the preterm infant (essential fatty acids—LNA and ALA—and their derivatives DHA and ARA) were selected. Total fat loss after 24 h was considered as a reference. Some fatty acids were not lost in a higher proportion than total fat, as in the cases of 18:1n-9 (0.5% ± 0.1%, *p* = 0.15), LNA (0.7% ± 0.1%, *p* = 0.25) and DHA (0.6% ± 0.1%, *p* = 0.56). However, in other cases, losses of some fatty acids were significantly greater than total fat loss, as with ALA (1.6% ± 0.3%, *p* = 0.02) and ARA (1% ± 0.2%, *p* = 0.02). In all cases, the magnitude of the loss was small after 24 h (Table 2). In Table 3 we presented curvilinear regression models predicting losses after 24 h of these selected fatty acids.

## 4. Discussion

How much HM fat is lost during continuous enteral feeding, and when this loss occurs during the infusion, is controversial. In addition, there are no data in the literature about what is exactly lost, in terms of individual fatty acid losses. To answer these questions, we have introduced three different elements in our experimental design, compared to previous studies:

### 4.1. Our Total Experiment Time Was Longer (up to 24 h of Infusion), Although We Still Included Intermediate Times

Our first objective was to determine whether the previously reported fat losses percentages could be related, at least to a certain degree, with the duration of the experiments. Some authors have suggested losses were higher only after 4 h [17] or even after 8 h of infusion [18]. However, another recent article suggested that losses appear to be higher at the beginning of the infusion [15], although the experiments in that particular study did not last more than 60 min. Our results are consistent with this last report and show that the duration of the feeding affects overall fat delivery efficiency. Significant fat loss in the first 30 min of infusion suggests that binding sites in the tubes become saturated at the beginning of the infusion. Later, percentage of fat loss is smaller maybe because there are not as many available binding sites. Thus, after some time of infusion, fat delivery efficiency increases, even though infusion flow velocity remains the same. Experiment duration has varied in previous studies but, to our knowledge, it has never been longer than 8 h. However, feeding tubes are changed every 48–72 h in real practice [23]. Thus, we designed a study over a 24-h period to mimic longer infusion times, which is closer to actual practices. We did not prolong to 48–72 h to avoid potential fatty acid oxidation of the breast milk during the experiment. As we used the same pooled milk during the whole experiment, fatty acid oxidation could have limited our conclusions, because the pre-infusion milk would have become somewhat “different” during the experiment. We can assure that in our case this change in the milk fatty acid composition did not occur because the milk fatty acid profile remained unchanged at time zero, mid and end of the study day. The velocity of milk flow is another important factor associated with fat loss. The lower the velocity, the higher the losses [24]. We chose a very slow flow (2 mL/h) to reproduce a worse-case-possible real scenario (less than 2 mL/h would represent trophic feeds for many preterm infants). However, we adhered to the best standard of care, and we also implemented some proven preventive measures: an upward tip position and gentle homogenization of the milk before infusion, to minimize the loss as we would do in our patients [8,13].

### 4.2. Direct Measurement of Adhered Fat to Nasogastric Tubes Instead of a Pre- and Post-Infused Milk Analysis

We recovered the fat remaining in the tube after the HM infusion and then, we determined the fatty acid composition on these samples. On the other hand, in previous studies, the milk coming out of the tubes was collected and then, the percentage of fat lost was calculated knowing the composition of the milk going in. We believe direct determination is more accurate than calculation. Moreover, we have proved this method is feasible. So far, no clinical studies involving real patients have been done. Therefore, the ultimate clinical relevance of fat loss during continuous enteral nutrition has never been reported. Our method could be used in future clinical studies, to measure fat loss affecting real patients, collecting feeding tubes after having been used in real patients, instead of recreating clinical practices in experimental conditions. Doing so, we could eventually relate fat losses with clinical outcomes.

### 4.3. Outcome Variables (not only Total Fat but also Individual Fatty Acids)

To our knowledge, this is the first report of the fatty acid composition of HM fat loss during continuous enteral nutrition. We speculate that the more fluid a fatty acid, the more easily it flows through the tube. Saturated and longer chain fatty acids were the more retained fatty acids over short infusion times (which would be the beginning of the continuous infusion in a real patient). This finding could be related to the fatty acid structure, which determines its fluidity [25]. Unsaturated fatty acids have lower melting points than saturated fatty acids of the same length. In other words, saturated fatty acids are less fluid. Also, the longer a fatty acid is, the less its fluidity.

This study has some limitations. We only measured fat adhered to the feeding and connecting tubes but not to the syringes. Nevertheless, the capacity of the syringe is greater, and contact between the milk and binding sites of its surface is less likely, so we think its effect would be neglectable. Only one flow rate (2 mL/h) was tested. We cannot extrapolate our results directly to other flow speeds. However, we assumed that at higher velocities losses would be less based on previous studies. Finally, we did not run milk for more than 24 h, although feeding tubes are used for longer times before being replaced in real patients. We do not know what the actual fat delivery would be after 48–72 h using the same feeding tube. However, our regression model suggests that as of 12 h of infusion the losses remain somewhat constant and low.

According to our results, the clinical significance of HM fat loss after 24 h of continuous infusion seems to be trivial, both quantitatively and qualitatively. When fat is oxidized, every gram of it produces 9 kcal. Considering our results, if an infant is fed 120–150 mL/kg/day in continuous enteral infusion, fat loss would mean 0.2–0.3 kcal/kg/day. This represents around 0.2–0.25% of the total caloric intake recommended for a preterm infant on enteral nutrition. Regarding quality does not seem to be clinically significant either. As stated previously, LCPUFA delivery to the preterm infant is insufficient following current nutritional recommendations [26]; but this problem does not appear to be worsened significantly by additional fat losses during continuous enteral nutrition. Mean HM fat content is 3.2 g–3.6 g/100 mL [27]. Worldwide, DHA and ARA fatty acids represent 0.32% ± 0.22% and 0.47% ± 0.13% of HM fat, respectively [28]. Fetal accretion rates are 95 mg of ARA and 42 mg of DHA per day during the last five weeks of gestation [29]. Thus, losses in continuous enteral feeding over 24-h infusion in a 1.5 kg infant would lead to a daily loss of 0.1–0.15 mg of DHA and 0.3–0.4 mg of ARA.

## 5. Conclusions

We conclude that continuous enteral feeding over 24 h resulted in no substantial loss of human milk fat. Therefore, feeding over a 24-h period does not appear to be a barrier to the delivery of fatty acids, including DHA and ARA fatty acids.

## Figures and Tables

**Table 1:** The fatty acid composition of the pooled human milk used in this experiment.

Fatty Acids	mg/100 mg of Fat
6:0	0.2 ± 0.10
8:0	0.3 ± 0.10
10:0	1.4 ± 0.20
12:0	5 ± 0.30
14:0	5.2 ± 0.10
15:0	0.2 ± 0.02
16:0	21.5 ± 0.20
17ai	0.1 ± 0.01
16:1t	0.3 ± 0.03
16:1 n7	1.3 ± 0.10
17:0	0.2 ± 0.02
18:0	8.3 ± 0.20
18:1 n9	40.6 ± 0.30
18:1 n11	1.7 ± 0.10
18:2 n6 (LNA)	11.6 ± 0.20
20:0	0.1 ± 0.02
18:3 n3 (ALA)	0.3 ± 0.05
20:1 n9	0.3 ± 0.10
20:3 n6	0.15 ± 0.10
20:4 n6 (ARA)	0.2 ± 0.03
22:6 n3 (DHA)	0.25 ± 0.02
Traces (<0.2%)	0.85 ± 0.20
∑SFA	42.6 ± 0.60
∑MUFA	44.8 ± 0.30
∑PUFA	12.5 ± 0.30
∑SMCFA	12.4 ± 0.70
∑LCFA	86.4 ± 0.50
∑VLCFA	1.2 ± 0.20

ALA: α-linolenic acid; LNA: linoleic acid; AA: arachidonic acid; DHA: docosahexaenoic acid; SFA: saturated fatty acid; MUFA: monounsaturated fatty acid; PUFA: polyunsaturated fatty acid; SMCFA: short-medium chain fatty acids; LCFA: long-chain fatty acid, and VLCFA: Very long chain fatty acid.

**Table 2:** Percentage of fatty acid loss (% of the total amount of FA initially infused that was recovered from the tube after the infusion time).

Fatty Acid	30 min	4 h	12 h	24 h
6:0	80.4 ± 13.2	8.3 ± 2.90	2.0 ± 0.30	1.2 ± 0.20
8:0	26.4 ± 0.60	4.5 ± 0.80	1.7 ± 0.10	1.1 ± 0.30
10:0	23.8 ± 8.20	5.5 ± 1.10	2.4 ± 0.40	1.5 ± 0.40
12:0	9.5 ± 1.80	2.4 ± 0.60	1.1 ± 0.10	0.8 ± 0.10
14:0	9.0 ± 0.01	1.3 ± 0.40	0.5 ± 0.10	0.4 ± 0.10
15:0	13.6 ± 3.80	2.0 ± 0.50	0.6 ± 0.20	0.4 ± 0.10
16:0	24.0 ± 1.60	2.9 ± 0.40	1.0 ± 0.10	0.6 ± 0.10
16:1 n7	8.0 ± 2.00	2.1 ± 0.80	1.1 ± 0.10	0.8 ± 0.20
17:0	23.4 ± 2.00	2.8 ± 0.50	1 ± 0.10	0.6 ± 0.10
18:0	31.4 ± 2.10	3.5 ± 0.60	1.2 ± 0.10	0.7 ± 0.10
18:1 n9	5.6 ± 1.80	1.3 ± 0.50	0.6 ± 0.10	0.5 ± 0.10
18:1 n11	4.7 ± 2.00	1.1 ± 0.50	0.5 ± 0.10	0.4 ± 0.10
18:2 n6 (LNA)	7 ± 2.10	1.8 ± 0.70	0.9 ± 0.10	0.7 ± 0.10
18:3 n3 (ALA)	10.5 ± 2.60	3.8 ± 1.80	2.2 ± 0.30	1.6 ± 0.30
20:0	64.4 ± 5.20	10.2 ± 2.10	3.4 ± 0.50	2.1 ± 0.60
20:3 n6	9.5 ± 4.20	2.5 ± 1.00	1.3 ± 0.20	0.9 ± 0.10
20:4 n6 (ARA)	11.6 ± 3.60	3.0 ± 1.00	1.5 ± 0.20	1.0 ± 0.20
22:6 n3 (DHA)	8.4 ± 2.10	1.6 ± 0.80	0.8 ± 0.20	0.6 ± 0.10
∑SFA	30.6 ± 2.60a	2.9 ± 0.40	1.1 ± 0.10b	0.7 ± 0.10
∑MUFA	5.8 ± 1.30a	1.3 ± 0.50	0.6 ± 0.10b	0.5 ± 0.10
∑PUFA	7.5 ± 2.00	1.9 ± 0.70	1.0 ± 0.10	0.7 ± 0.10
*p*-value *	0.015	0.06	0.02	0.08
∑SMCFA	13.8 ± 6.10	2.4 ± 0.50	1.0 ± 0.10	0.7 ± 0.10
∑LCFA	14 ± 4.50	2.0 ± 0.40c	0.8 ± 0.01d	0.6 ± 0.10e
∑VLCFA	23.5 ± 2.50	3.7 ± 0.50c	1.4 ± 0.20d	0.9 ± 0.20e
*p*-value *	0.09	0.02	0.007	0.04

Results are expressed as mean % ± standard deviation. Distributions were compared for degree of unsaturation and length chain at every time point by Kruskal-Wallis test (* *p*-value is presented). A, b, c, d and e, indicate significant differences in the one to one post-hoc analysis (Dunn-Bonferroni test). ALA: α-linolenic acid; LNA: linoleic acid; AA: arachidonic acid; DHA: docosahexaenoic acid; SFA: saturated fatty acid; MUFA: monounsaturated fatty acid; PUFA: polyunsaturated fatty acid; SMCFA: short-medium chain fatty acids; LCFA: long-chain fatty acid; VLCFA: Very long chain fatty acid.

**Table 3:** Curvilinear regression models to predict loss of some selected fatty acids after 24 h of HM infusion. ALA: α-linolenic acid; LNA: linoleic acid; AA: arachidonic acid; DHA: docosahexaenoic acid.

Fatty Acids	Coefficient of Determination (R²)	Curvilinear Model Equation	*p*-Value
16:0	0.99	y = −0.007 + 11.98/x	<0.0001
18:1 n9	0.86	y = 0.477 + 2.562/x	<0.0001
18:2 n6 (LNA)	0.87	y = 0.732 + 3.143/x	<0.0001
18:3 n3 (ALA)	0.85	y = 1.969 + 4.285/x	<0.0001
20:4 n6 (ARA)	0.87	y = 1.177 + 5.257/x	<0.0001
22:6 n3 (DHA)	0.92	y = 0.494 + 3.985/x	<0.0001
